# The routine use of pediatric airway exchange catheter after extubation of adult patients who have undergone maxillofacial or major neck surgery: a clinical observational study

**DOI:** 10.1186/cc2956

**Published:** 2004-09-22

**Authors:** Levent Dosemeci, Murat Yilmaz, Arif Yegin, Melike Cengiz, Atilla Ramazanoglu

**Affiliations:** 1 Assistant Professor, Department of Anesthesiology and ICU, Akdeniz University Hospital, Antalya, Turkey; 2 Specialist, Department of Anesthesiology and ICU, Akdeniz University Hospital, Antalya, Turkey; 3 Professor, Director of Department of Anesthesiology and ICU, Akdeniz University Hospital, Antalya, Turkey

**Keywords:** airway exchange catheter, difficult intubation, maxillofacial surgery, neck surgery, reintubation

## Abstract

**Introduction:**

We conducted the present study to determine the usefulness of routinely inserting a pediatric airway exchange catheter (PAEC) before tracheal extubation of adult patients who had undergone maxillofacial or major neck surgery and have risk factors for difficult reintubation.

**Methods:**

A prospective, observational and clinical study was performed in the 25-bed general intensive care unit of a university hospital. Thirty-six adult patients who underwent maxillofacial or major neck surgery and had risk factors for difficult reintubation were extubated after insertion of the PAEC.

**Results:**

Four of 36 (11.1%) patients required emergency reintubation after 2, 4, 6 and 18 hours after tracheal extubation, respectively. Reintubation of these patients, which was thought to be nearly impossible by direct laryngoscopy, was easily achieved over the PAEC.

**Conclusion:**

The PAEC can be a life-saving device during reintubation of patients with risk factors for difficult reintubation such as laryngeo-pharyngeal oedema due to surgical manipulation or airway obstruction resulting from haematoma and anatomic changes. We therefore suggest the routine use of the PAEC in patients undergoing major maxillofacial or major neck surgery.

## Introduction

Maxillofacial and major neck surgery has a considerable risk for postoperative laryngo-pharyngeal oedema and airway obstruction due to surgical manipulation or haematoma [1]. When patients undergoing these operations develop laryngeal oedema or airway obstruction and require reintubation after they have been extubated, reintubation may be very difficult or impossible through laryngoscopy because of the characteristics of these operations such as mandibular fixation with an archbar or as a result of anatomical changes. Extubation of a patient with risk factors for difficult tracheal reintubation is approached with concern, even in the experienced hands of the anaesthesiologist and critical care physician. Although all of the criteria used to predict successful extubation are generally satisfactory before extubation, none predict the adequacy of the airway once the endotracheal tube (ETT) has been removed [2].

Hence, acute respiratory distress can develop after extubation and mandate emergency tracheal reintubation. Mask ventilation and tracheal intubation may be difficult or impossible. Considerable time and an experienced physician are needed to secure a difficult airway with the use of alternative methods such as fibre-optic bronchoscope, retrograde] intubation or cricothyroidotomy. Re-establishing the airway in these patients can be extremely challenging, and often results in considerable morbidity and mortality [3]. In the study by Loudermilk and colleagues [2], the advantages of the use of a pediatric airway exchange catheter (PAEC) inserted before tracheal extubation of adult patients with a known or expected difficult airway were well shown. However, the routine use of PAEC as a rescue for reintubation after maxillofacial surgery has not been reported.

**Figure 1:**
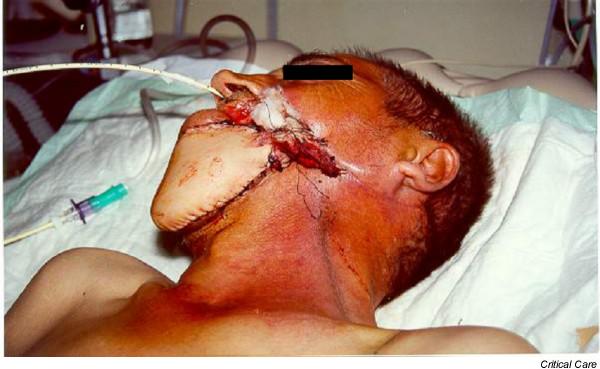
A patient who had undergone maxillofacial reconstructive surgery was extubated with the use of a pediatric airway exchange catheter (PAEC) in the intensive care unit. The PAEC was left in place for 6 hours, and the patient did not require reintubation after the PAEC had been removed.

The aim of this study was to determine the usefulness of routinely inserting the PAEC before tracheal extubation of adult patients undergoing major maxillofacial or neck surgery (Fig. 1).

## Methods

### Patients

Thirty-six patients admitted to our intensive care unit (ICU) after maxillofacial or major neck surgery between January 2001 and May 2002 were routinely extubated with the use of a no. 11 PAEC (Cook Critical Care, Bloomington, IN), with the approval of the Institutional Review Board. Patients included in the study consisted of 13 post-operative patients with maxillofacial trauma, 14 patients who had undergone neck surgery (5 with hugely enlarged thyroid gland or tumor and 9 with larynx or tongue cancer), and 9 patients who had undergone maxillofacial cancer surgery.

Written consent for publication of the photos of the patients was obtained.

### Technique

A no. 11 PAEC is 83 cm in length and has a 4 mm external diameter and a 2.3 mm internal diameter with a hollow lumen. It is semi-rigid and made of radio-opaque polyurethane; there are six sideports in the distal 3 cm of the catheter. The patients were extubated when they became conscious and had normal body temperature and normal blood gases with an inspired oxygen concentration (FiO_2_) of 0.4, a positive end expiratory pressure of less than 5 cmH_2_O and pressure support of less than 8 cmH_2_O. In addition, the haemodynamic status of the patients had to be stable before the decision to extubate was made. The PAEC was carefully inserted through the existing ETT before extubation, avoiding carinal irritation by placing it at the same depth as the ETT tip (20–22 cm orally or 27–30 cm nasally). The PAEC was not inserted against a resistance. After the ETT had been removed and the PAEC had been secured, humidified oxygen with a low flow of 1–2 l/min was insufflated via the lumen of the PAEC. Signs of respiratory failure and tolerance were also assessed. The PAEC was removed when it became clinically apparent that the need for tracheal reintubation was unlikely. We considered the ability of patients to manage secretions including cough and swallow functions in making the decision about extubation of the PAEC. A stable O_2 _saturation and the extent of surgery were also important factors in this decision. The timing of removal of the PAEC was therefore different depending on various characteristics of patients and surgery. When patients failed to respond to tracheal extubation, the PAEC was used to facilitate the reintubation.

## Results

Twenty-eight patients (77.8%) were men, and 8 (22.2%) were women. Ages ranged from 19 to 76 years, with a mean age of 52.6 ± 10.8 (all results are means ± standard deviation) years. An oral ETT was in place in 18 patients (50%) and a nasal ETT in 18 (50%). All patients had a cuff leak test before tracheal extubation. The median duration of endotracheal intubation after the operations was 1.2 days (range 2 hours to 10 days). After tracheal extubation with the PAEC, 4 of 36 patients (11.1%) required reintubation (Table 1). The reintubation of these four patients, who are discussed in detail as case reports below, was achieved over the PAEC and was easily accomplished on the first attempt without the need of an alternative method. We used the assistance of laryngoscope during the reintubation of two patients in whom the PAEC had been inserted orotracheally. In the other 32 patients who did not require reintubation, the PAEC was kept in the trachea for between 4 and 24 hours (mean 10.4 ± 4.2 (all results are means ± standard deviation) hours) and none of them required reintubation after the PAEC had been removed. Thirty-one patients had nasogastric tubes at the same time. The PAEC was well tolerated in 34 of 36 patients (94.4%). Two patients tried to remove the PAEC; they were therefore sedated for a few hours. We did not give any sedative drugs to the patients who could tolerate the PAEC. No adverse events were observed while the PAEC was being kept in the trachea.

### Case 1

The reason for reintubation of this male patient, who had undergone radical neck surgery for cancer and had been intubated easily by direct laryngoscopy before the operation, was excessive surgical bleeding and haematoma, which developed 2 hours after extubation. The patient was immediately taken to the operating room. He could not be ventilated effectively by bag-valve-mask during the induction of anaesthesia (fentanyl 2 μg/kg, propofol 2 mg/kg, vecronium 0.1 mg/kg) and his oxygen saturation decreased to 85%. He was reintubated orally over the PAEC with the assistance of a laryngoscope within a few seconds by using an 8 mm ETT. During observation with a laryngoscope, reintubation of this patient by direct laryngoscopy was thought to be nearly impossible because the glottis could not be seen as a result of the anatomic abnormality caused by haematoma. He was extubated again using the PAEC 24 hours after his second operation; the PAEC was removed again 6 hours after insertion.

### Case 2

The second patient (a male), who had also undergone neck surgery (unilateral dissection), was intubated with difficulty using a Fasttrach (intubating laryngeal mask airway) because of anatomical abnormalities, which developed as a result of previous operations and radiotherapy. He was extubated 4 hours after the operation in accordance with the criteria mentioned above. However, he required emergency reintubation 18 hours after extubation because he suffered acute respiratory distress following aspiration and bronchospasm. We found out from the history obtained from his relatives that the patient had already had a swallowing disorder before the operation and suffered from aspiration. Thus, we prolonged the presence of the PAEC. Reintubation of this hypoxic patient was urgently achieved over the PAEC with the assistance of a laryngoscope using a 7.5 mm ETT under sedation and neuromuscular relaxation. During laryngoscopic observation we could not see the glottis. In this patient, a surgical tracheotomy was performed later because of recurrent aspiration and the need for tracheal suction.

### Case 3

**Figure 2:**
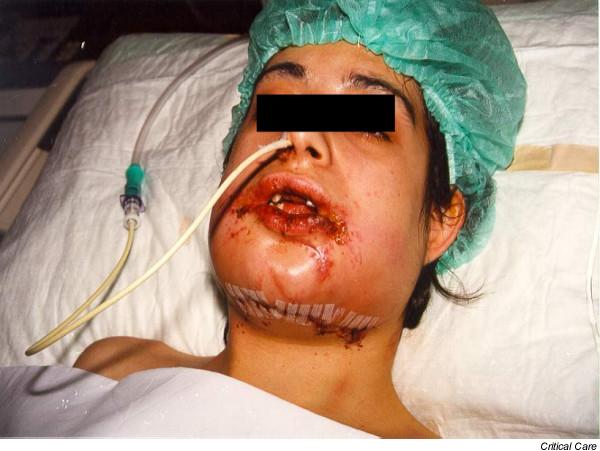
A patient who underwent maxillofacial surgery due to trauma, presented as case 3 in the text. She was extubated with the use of the pediatric airway exchange catheter (PAEC), and required reintubation after 6 hours of extubation. This was easily achieved over the PAEC without cutting the archbar.

This patient (a female) was admitted to the ICU after she operation for maxillofacial trauma. She had been intubated nasally by direct laryngoscopy using a Magill forceps; she could not open her mouth after the operation because of inter-maxillary fixation (Fig. 2). Six hours after extubation her arterial CO_2 _pressure increased, and she became confused as a results of hypoxaemia. She was reintubated nasally with a 7 mm ETT over the PAEC, with intravenous midazolam 0.05 mg/kg and fentanyl 1 μg/kg, without cutting the archbar. She was extubated with the use of the PAEC 2 days after her reintubation, and the PAEC was left in place for 8 hours. She did not need intubation again after the PAEC had been removed.

### Case 4

The fourth patient, a male, underwent maxillofacial reconstructive surgery for cancer after he had been intubated nasally over a flexible bronchoscope because of anatomical abnormalities in the oral route. He became hypoxic 4 hours after his extubation and required immediate reintubation. A serious pharyngeo-laryngeal oedema was thought to be the reason for hypoxia. His reintubation was easily achieved over the PAEC, with intravenous midazolam 0.05 mg/kg. He was extubated with the use of the PAEC 3 days after reintubation, and the PAEC was left in place for 6 hours. He did not require intubation again after removal of the PAEC.

## Discussion

During the perioperative period, serious respiratory events due to inadequate airway management can develop, which can cause severe brain damage or death. Rosenstock and colleagues [4] reported that 60 of 284 complaints filed at the National Board of Patients' Complaints in Denmark over a period of 4 years were associated with perioperative respiratory complications, 50% of which resulted in death. Adverse outcomes associated with respiratory events constituted the single largest class of injury in the American Society of Anesthesiology Closed Claims Study (522 of 1541 cases; 34%). Death or brain damage occurred in 85% of these cases. Three mechanisms of injury accounted for three-quarters of the adverse respiratory events: inadequate ventilation (38%), oesophageal intubation (18%) and difficult tracheal intubation (17%) [5].

In previous studies, reintubation rates of 5–19% have been reported in surgical ICU patients [6-8]. In our study, 11% of the patients required reintubation because of surgical bleeding, pharyngo-laryngeal oedema, aspiration, and inability to manage secretions. The reintubation risk of our study patients was higher than general ICU patients because they had high risks in terms of airway obstruction due to surgical manipulation. Patients who are expected to have a difficult airway may remain intubated longer than necessary, simply for fear of the inability to reintubate. Before the use of the PAEC in our clinic, we usually restricted extubation of patients who had undergone maxillofacial surgery and were at risk of difficult reintubation to the daytime, when experienced physicians were available, rather than during the night, to provide safer conditions. Prolonged tracheal intubation not only increases the risk of complications but is also expensive because it requires respiratory therapy and more extensive monitoring [9].

The PAEC is a long, flexible and hollow tube designed to facilitate the exchange of an *in situ *ETT. The primary use of the PAEC (adult size, 16–18 F) has been as a tube exchanger in the critical care setting. It has been also used before the extubation of patients with a known difficult airway [10]. In the study of Loudermilk and colleagues [2], the use of the PAEC in 40 patients with risk factors for difficult reintubation, including a history of previous difficult intubation, airway edema secondary to surgical manipulation or volume resuscitation, morbid obesity, and an immobilized or unstable cervical spine, was well described. They reported that 3 of 40 patients (8%) had been easily reintubated with the use of PAEC, which is a reintubation rate similar to our results. Although our findings are similar to those in the study of Loudermilk, our study population consisted of a specific surgery group and we used PAEC as a routine procedure in this group without considering whether the patients had previously been intubated with difficulty.

Various methods have been used to facilitate the reintubation of these patients such as a fibre-optic bronchoscope [11], rigid ETT guides [12] and retrograde intubation. When all of these methods fail, an urgent cricothyroidotomy or tracheotomy may be the only solution. The PAEC offers several advantages over these alternatives: first, it provides a method for the continuous administration of oxygen; second, it can be used as a stylet for tracheal reintubation; and third, it provides a method of ventilating the patient (jet ventilation) [13].

In patients whose reintubation was considered a risk and who were known to present difficult tracheal reintubation, elective tracheotomy has even been performed in many institutions [2]. Besides, there have been many cases reported who have undergone tracheotomy because of airway obstruction or other respiratory pathologies after neck surgery [14,15]. Intraoperative tracheotomy is a safe route to secure the airway in the postoperative period in patients undergoing maxillofacial or major neck surgery. However, tracheotomy is a considerably invasive method and can lead to serious complications including bleeding, pneumothorax, infection and tracheal stenosis. Furthermore, only about 10% of the patients undergoing maxillofacial or neck surgery require reintubation after their operations, and most of these patients can be extubated later. This means that performing the tracheotomy routinely is not necessary in most of these cases. However, sometimes tracheotomy can be unavoidable in a selected group, especially when the patients are expected to need prolonged mechanical ventilation or are at great risk of reintubation because of severe airway obstruction. Thus, both methods can be considered depending on patient characteristics. In addition to the operative factors, the patients should be meticulously evaluated before the operation in terms of respiratory capacity, neurological status and co-morbid factors. However, there are no strict criteria for a decision on tracheotomy or a trial extubation. For example, our case 2 would have benefited from an intraoperatively performed tracheotomy. Fortunately, we were able to reintubate this patient easily over the PAEC, and then decide to perform the tracheotomy.

Although the PAEC is rigid enough to facilitate tracheal reintubation, not all patients' tracheas may be easily reintubated. Forceful insertion of the ETT should be avoided to minimize trauma to vital airway structures and to avoid kinking the PAEC. Direct laryngoscopy may also relieve the obstruction and identify its cause. We also used the assistance of the laryngoscope during the reintubation of two patients over the PAEC both to facilitate the intubation and to evaluate the anatomical structure of the upper airway with regard to the possibility of laryngoscopy. Gentle rotation of the ETT while trying to insert it may release the tip [16]. The PAEC should never be inserted against a resistance. Although the tip of the PAEC is rounded and blunt, perforations of the tracheo-bronchial tree during the insertion of these catheters have been reported [17,18].

In a study of patients requiring tracheal reintubation, 87% (34 of 39) required reintubation within the first 4 hours after extubation [19]. In our series, one patient required reintubation 2 h after extubation, two reintubations occurred within 6 h and the other 18 hours after extubation. This finding shows that the need for reintubation later than 4 hours after extubation is not rare. As it is impossible to know at what time patients may develop respiratory distress, the timing of removal of the PAEC can be decided only on an individual basis. We have no data to determine the optimal period for which the PAEC should be left in place. Potential complications of the prolonged use of PAEC are airway trauma and aspiration caused by incomplete glottal closure. One of our patients who underwent neck surgery and required reintubation after 18 hours of extubation aspirated before the reintubation, but this patient had already had swallowing dysfunction due to radiotherapy before the operation. We therefore considered that the aspiration was not associated with PAEC only, although it could have contributed to the development of aspiration. Besides, the presence of the PAEC in the trachea can cause the retention of tracheal secretion by inhibiting effective coughing, especially in patients with chronic pulmonary disease, smokers, or patients who stayed immobile for a long time before surgery. In these conditions, the PAEC should be left in place for as short a duration as possible.

## Conclusion

The routine use of a PAEC in patients who have undergone maxillofacial or major neck surgery facilitates reintubation when necessary, and can be a life-saving method. It allows a safer trial of tracheal extubation and therefore can shorten the duration of intubation. We suggest that after these surgical procedures a PAEC be used routinely before tracheal extubation because it is difficult to predict which patients will require reintubation.

## Key messages

• 	The PAEC is a long, flexable and hollow tube designed to facilitate the exchange of an in-situ endotracheal tube.

• 	The routine use of the PAEC in patients who underwent maxillofacial or major neck surgery facilitates the reintubation when necessary, and can be a life-saving method.

## Competing interests

The authors declare that they have no competing interests.

## Abbreviations

ETT = endotracheal tube; ICU = intensive care unit; PAEC = pediatric airway exchange catheter

## Figures and Tables

**Table 1:** Demographic data, duration of use of endotracheal tube and pediatric airway exchange catheter, and reintubation ratio

Parameter	Value (*n *= 36)
Sex (F/M)	28/8 (77.8%/22.2%)
Age (years)	52.6 ± 10.8 (range 19–76)
Pathology	
Maxillofacial trauma	13 (36.1%)
Neck surgery	14 (38.8%)
Maxillofacial cancer surgery	9 (25.0%)
Endotracheal tube, oral/nasal	18/18 (50%/50%)
Duration of endotracheal intubation (days)	2.8 ± 1.6 (range 0.1–10)
Reintubation ratio	4/36 (11.1%)
Duration of PAEC^a ^(h)	10.4 ± 4.2 (range 4–24)

PAEC, pediatric airway exchange catheter.

^a^In 32 patients who did not require reintubation.
